# Book Reviews

**Published:** 1825-08

**Authors:** 


### CRITICAL ANALYSIS
OF
### ENGLISH AND FOREIGN LITERATURE,
RELATIVE TO THE VARIOUS BRANCHES OF
### Medical Science.

Quæ laudanda forent, et quæ culpanda, vicissim

Illa, prius, cretâ; mox hæc, carbone, *notamus*.—PERSIUS.

### DIVISION I.
### ENGLISH.

ART. I.—*An Essay on Egyptian Mummies; with Observations on the Art of Embalming among the ancient Egyptians.* By A. B. GRANVILLE, M.D. F.R.S. &c. &c. Read April 14th, 1825, before the Royal Society.

THE author of the above Essay having kindly presented us with a copy, we hasten to give our readers an account of the interesting researches it contains; which we are the more disposed to do, since some time must yet elapse before the public will have an opportunity of perusing it in the Transactions of the learned body before whom it was read.

Dr. GRANVILLE, in this inquiry, has not confined himself to a mere description of a solitary specimen of the mummy, but has given a comprehensive view of the whole of his subject, which admits of a very easy division into three principal parts: the first containing the history of what was previously known regarding Egyptian mummies; followed, secondly, by a description, both external and internal, of the specimen in question, including some questions highly interesting to the natural history of man; and, thirdly, an account of the probable mode of making these preparations, and of the substances employed upon these occasions, terminating in a recapitulation of the various processes requisite for the preparation of the most perfect kinds of mummies. This, though not exactly the order followed by our author, is the one which we have selected as enabling us to give in the smallest compass the substance of what is contained in upwards of forty quarto pages; a plan which will, we conceive, be more acceptable than mere extracts, since the subject is one but imperfectly understood, either by the profession or the public at large.

The first account of Egyptian mummies which is to be found in the records of learned bodies in this country, is contained in a paper by Dr. HADLEY, published in the Transactions of the Royal Society in 1764, but does not appear to have added much to what was then known on the subject. The specimen he examined had not the smallest vestige of soft parts; a bulbous root, probably an onion, was found attached to one of the heels by fillets and pitch; the bones were all more or less brittle, and some of them separated into splinters under examination. In 1794, BLUMENBACH presented to the same Society an account of three small and one large mummy, which he had opened when in London; but in none of these did he discover any thing but mere bones: nay, in one there was nothing but a mass of bandages, strongly impregnated with resinous substances. Neither does it appear that the learned inquirers on the continent had been more successful; and our author mentions several who had investigated the subject, with no happier results. Long, however, before Hadley or Blumenbach had directed their attention to this inquiry, ROUELLE, the French chemist, and CAYLUS, had treated the subject with great precision; and the former, in a paper published in the Memoirs of the French Academy of Sciences, has described several chemical operations to which he subjected his mummies, in order to ascertain the nature of the ingredients employed by the Egyptian embalmers; but he by no means arrived at any satisfactory conclusion. With regard to the anatomical state of the mummies, he adds nothing to our previous knowledge.

Of Professor HEYNE's papers, published in the Transactions of the Royal Society of Gottingen, and of a paper by Professor Gmelin, containing the result of various chemical experiments made upon one of those mummies, we need only observe, that they by no means confirm Rouelle's account; though, at the same time, they lead to no more satisfactory explanations. Professor Heyne remarks, that not only had the viscera been taken out, but the muscles and all the soft parts had been removed by accurate dissection, in the specimens examined by him.

The sçavans who accompanied the French expedition to Egypt did not neglect the opportunity thus afforded them of inquiring into this subject. The number of mummies discovered by them was prodigious, and, although they were in different degrees of preservation, still they appeared to contain little more than the skeleton; nor do Baron Larrey's Memoirs, which are chiefly intended to determine the identity of the present race of Copts with the aboriginal Egyptians, by a comparative examination of the crania of several mummies collected in the desert of Saqquarah, and those of the modern Copts from a cemetery in Alexandria, go in any degree to settle the question, since the mummies of Saqquarah are allowed to be very inferior to those of Upper Egypt.

Besides the information thus derived from the writings of different authors, Dr. Granville presents us with the testimonies of Sir E. Home, Dr. Baillie, Mr. Brodie, and Mr. Clift, to show that, though these gentlemen had directed their attention at various times to the examination of mummies, they made no very new or interesting discoveries, in consequence of the imperfect condition of the specimens that fell under their observation. Of these, one only appears, from Sir E. Home's account, to have been of a more perfect kind; but its remains seem to have been neglected, and left to destruction in some of the vaults of the College. A few remarks, made also by Sir E. Home, on a mummy brought from Thebes by the late Captain Kennet, follow next in order; but here no internal examination was permitted.

The dimensions of this mummy are given by our author; but we must now hasten to the history and examination of the specimen which forms the most interesting portion of this paper, omitting some observations on the head and right arm of a male mummy, brought from the neighbourhood of Tripoli in Africa, as not immediately connected with the inquiry before us, and having a regard to the limited space which we are enabled to devote to any one subject.

The mummy which Dr. Granville has examined was presented to him by Sir A. Edmonstone, and was purchased by him at Gournou in March 1819, from one of the inhabitants of the sepulchral excavations on the side of the mountain, at the back of which are the celebrated tombs of the kings of Thebes. It had no outer case, but the surface of the case was in beautiful and perfect condition; and it is remarked by Sir A. Edmonstone, that those mummies which have an outer case are folded externally with more care than the one in question, and that the outer folds are ornamented with variegated stripes of linen. The single case of this mummy was made of sycamore wood, two inches thick, consisting of two equal portions anterior and posterior, so that it could be stood on the feet, and these were fastened by pegs of the same wood. Externally, it is covered with hieroglyphics, on a deep orange ground, highly varnished; internally, the surface is striped horizontally, excepting at the sides, where they are perpendicular: these are white and yellow alternately. The length of this case is six feet five-tenths of an inch; and its circumference, at three different points, was respectively five feet two inches,—four feet eleven inches and three-tenths,—and three feet eight inches and six-tenths. The mummy itself was covered with cerecloth and bandages, most skilfully arranged, and applied with a neatness and precision that would baffle the skill of the modern surgeon. Every kind of bandage mentioned by the ancient writers was here met with. The precision of the folds and neatness of the turns, as well as the judicious selection of their size, length, and forms, excited the admiration of those who witnessed the operation. In many parts, where depressions or hollows were to be filled up, compresses were found; and each limb, each finger and toe, had a separate bandage next to the skin. The principal rollers appeared to be made of a compact, yet elastic linen, some of them four or five yards in length, without a stitch or seam. Some large square pieces were thrown round the head, thorax, or abdomen, which were found to alternate with the complete swathing of the whole body: they occurred four times, whilst the bandaging with rollers, &c. was repeated at least twenty times. All these bandages were covered by a roller, three and a half inches wide and eleven yards long, which ascended in graceful spirals to the head, and, descending again, was fixed to the breast: the end of this was fringed, and had some characters traced on it, one or two of which had corroded the linen, leaving the traces of their form. Besides the above, there was another bandage thrown over the head, brought in front of the chest, crossed there, and carried behind the back, again brought in front, returned backwards, and lastly stretched out before down to the feet, where it was crossed a third time: the shape, form, and position of the limbs, lay thus completely concealed. There was also a thick fold of linen, by no means neatly folded up, laid upon the face, covered by a layer of black bituminous substance, most effectually concealing the features.

Our author next proceeds to inquire of what substances these bandages were composed; and he decides that both cotton and linen were employed in the preparation, though HERODOTUS only mentions the former. The test by which he satisfies himself upon this point was to rub separate portions, first freed from all extraneous matter, with a rounded piece of glass or ivory; the linen will thus acquire a considerable lustre, but the cotton will only have the threads flattened. Dr. Granville submitted his specimens, so treated, to an experienced manufacturer, who confirmed his opinion.

The removal of the various envelopes of the mummy occupied upwards of an hour: it was then ascertained that the subject was a female, and no ventral incision, as described by Herodotus, had been practised to extract the viscera. The external parts of generation, totally free from hair, had been brought into close contact, but were readily recognised. The mammæ must have been large, for they extended as low as the seventh rib, against which they were pressed by the arms; but, when these were lifted, the breasts were raised without difficulty: the nipples and the areolæ were seen in a perfectly distinct manner. The head is closely shaved; the hair may be felt upon passing the hand over it, and appears to be brown. The eyelids were in close contact; and the cranium does not seem to have been disturbed externally. The teeth are seen perfectly white, and in a sound condition. The arms are crossed over the chest; the fore-arms directed obliquely upwards. The fingers of the left hand were bent inwards, the thumb being extended. There was no papyrus or other object of interest within the grasp,—only a lump of rags, dipped in the bituminous substance; neither were there any idols or papyri under the arm-pits. A few glass beads, of a blue and green colour, and some bugles, only were found between some of the rolls of the bandages, as if dropped in by accident. A portion of reddish clay, with characters painted on it, was also found in a situation to act as a compress in the inside of the left leg; and which our author conjectures to have been either a fragment of the wall of the chamber in which the mummy was made, or a piece of a case belonging to some other mummy. Here it was used to fill up a hollow; and Dr. Granville thinks that the same explanation will serve for the accidental presence of the beads.

The inferior extremities were brought into close contact at the knees and toes, the latter being fastened together. Numerous wrinkles were found on the integuments of the abdomen, denoting that its dimensions must have been considerable before death.

The general surface of the body was of a deep brown colour, and quite dry. In parts where the larger muscles lie, the surface felt quite soft, and they yielded slightly to pressure. The cuticle had been removed throughout, except at the extreme points of the fingers and toes; the nails, of a brown colour, being retained, but they were easily detached.

The dimensions of the mummy formed the next subject of inquiry, especially as the Egyptian has been assumed by BLUMENBACH as the type of a specific variety of the Ethiopian race. The height of this specimen was five feet seven-tenths of an inch. The dimensions of the various parts of the trunk and extremities is next given, which, upon comparison with those of the Venus de Medicis, as given by WINKELMAN, CAMPER, &c. prove to be as nearly as possible the same. But, continues our author, it is the female pelvis that presents the most striking difference in different races: thus, nothing can be more distinct than this part in the Negro, and the Caucasian or European race. In subjecting the pelvis of this mummy to this comparison, it was found to come nearer to the *beau ideal* of the Caucasian structure, than that of European women in general; equalling in depth, amplitude, and rotundity of outline, the Circassian form. The dimensions are then given; from which it appears that they are precisely in the proportion which the longest diameter bears to the shortest in the Venus, according to Camper,—viz. as 46 to 34; whereas, in the Ethiopian or Negro race, it is only as 39 to 27½. The same remarks apply with equal force to the head, in which the likeness it bears to the skull of the Georgian female, as represented in Blumenbach's "Decas tertia Craniorum," is very striking. This examination, therefore, tends to confirm CUVIER's opinion respecting the Caucasian origin of the Egyptians; and it has also been remarked by travellers, that whole families are to be found in Upper Egypt, the characters of whose head, face, and figure, resemble strongly those of the best mummies found in the hypogey of Thebes.

We now proceed to describe the dissection of this mummy. An incision having been made into the parietes of the abdomen below the ribs, down to the hip-bone, on each side, the whole of the integuments and muscles were removed, exposing the cavity. Here was found a portion of the stomach adhering to the diaphragm; the spleen, small and flattened; the left kidney, with its ureter and suprarenal capsule. This, together with the uterus and its appendages, were in their natural situation, exhibiting strong marks of having been diseased. Fragments of the intestines only could be found; among these were the cœcum, and its appendix vermiformis.

There were found lumps of a species of brittle resin, two or three pieces of myrrh in their natural state, and some larger lumps of a bituminous substance, mixed with argillaceous earth:

these seemed to have been forced up to fill the cavity, after the removal of the largest portion of the intestines, which appear to have been extracted through the anus in a very clumsy manner; this orifice being cut in various directions.

No traces of the right kidney or liver could be detected; but Dr. Baillie, who was present at one of the demonstrations, found the gall-bladder slightly lacerated, but otherwise perfect, with some remains of its duct, and of the peritoneal covering of the liver attached to it.

In removing the soft parts from the pelvis, the Doctor, and the gentlemen who witnessed the examination, were much struck with the remarkable degree of preservation of the muscles, admitting their being separated from each other, as in the dissection of a recent subject. The membranes and ligaments of the joints were equally perfect, permitting free motion of the thigh with the ilium.

The cavity of the thorax was examined, without disturbing the bones, by removing the diaphragm. The pericardium, which adhered to it, came away with it; the heart was found *in situ*, suspended by its great vessels; the lungs adhered, throughout their posterior surface, to the ribs, and were brought away in as perfect a state as could be effected.

The cranium was next sawn through horizontally, and it was ascertained that the brain had been removed through the nostrils; and it is not a little surprising how every vestige of the membranes investing the brain could have removed by these means: it could scarcely be done, but by some species of injection. A black resinous substance, in a small quantity, was found adhering to the inner surface of the occipital bone. Whatever the liquid was, it had evidently been employed quite hot, as it had partially burnt the superior part of the lambdoidal suture. The eyes do not appear to have been disturbed; the tongue was preserved, but there was not any piece of coin found, either above or under it, but a lump of rags dipped in pitch. The teeth did not present that peculiar cylindrical form of the incisors, said to be one of the characters of the Ethiopian race.

Dr. Granville next turns his attention to the age of the female under consideration, and to the disease of which she died; and it is one of the most curious circumstances connected with this inquiry, that, after the lapse of three thousand years, any thing like probability can be arrived at on these points; yet we conceive that our author makes out very satisfactorily that this female was about fifty or fifty-five years of age; that she had borne children; and died of ovarian dropsy, attended with structural derangement of the uterine system generally.

The first fact, that of the age, is proved by the peculiar degree of thinning in the centre of the osseous plates, which has been noticed by CHAUSSIER and others in the female skeleton, as an indication of their having borne children, and passed the fortieth year of their age, and which reaches its maximum at fifty-five. In this mummy, the thinning of the central portions of the ilium is so complete, that small fragments have come away in consequence of their having been so frequently touched by the incredulous.

Respecting the disease which destroyed her, our author observes, that the womb was larger than it is known to be at the age in question; that the ovarium and broad ligament on the right side were enveloped in a mass of diseased structure; the fallopian tube of the same side was perfectly sound, and beautifully preserved; whilst the contracted parietes of what appears to have been a large sac connected with the left ovarium, leaves no room to doubt the correctness of this explanation, which was strengthened by the concurring opinions of the late Dr. BAILLIE, Mr. WILSON, Mr. CARPUE, Mr. BROOKS, and others.

We now enter upon another subject of inquiry,—namely, in what manner these more perfect specimens of Egyptian mummies were produced. This leads to the consideration of how far the mummy in question agrees with the description of the ancient writers; and, finally, to an inquiry into the nature of the substances employed in their preparation.

In order to come to a decision on the last of these points, Dr. Granville enumerates some facts resulting from a close examination of his mummy, and then treating those facts both analytically and synthetically. The first fact is the brown tint of all the bandages. From experiments made with portions taken from several parts of the body, it was found that they had all been steeped in some vegetable solution, which, treated with gelatine, exhibited the presence of tannin, and which was corroborated by the taste of the infusion; from which it is inferred that the embalmers were acquainted with the antiseptic powers of astringent and bitter vegetable infusions. The second fact strengthens this inference, which is the brown appearance of the integuments, differing in no respect from prepared leather; and a question here arises whether the bark of the acacia, so plentiful in Egypt, was used for this purpose, or whether oak-bark was imported from Syria, where that tree grows in abundance? Neither of the above circumstances had ever been noticed before. The next fact is the appearance of minute saline crystals, found in almost every part of the external, and more so of the internal, parts of the body: at first these were not noticed; but, upon various portions of the mummy being exposed to the open air, in a room where a fire was kept, they became visible. This efflorescence was gently swept off with a brush, and subjected to various analyses; the result of which was, that it consists of nitrate of potash, carbonate, sulphate, and muriate of soda, with traces of lime; and, as these salts have not been found to form spontaneously either within or on the surface of preserved bodies, it follows that the body must have been immersed in a saline solution of a mixed kind. The presence of lime may be accounted for by supposing that the cuticle was removed by means of that substance; proving that the embalmers were aware that, in order to promote the absorption of liquid substances, particularly of tannin, the cuticle must be first removed. M. Royer has, among others, mentioned the presence of saline substances; but the origin of them has not been before hinted at. The fourth fact demanding our attention, is the presence of a resino-bituminous substance, found between some of the folds of the peritoneal membrane. On examination, it was ascertained that the bitumen was mixed with so great a proportion of wax as to make it plastic: it must either have been injected warm into the cavity of the abdomen, or the body must have been plunged into a liquified mixture of this kind, and kept for hours, or days, over a gentle fire. This latter explanation, which has been before surmised, our author considers as proved by the thoroughly impregnated state of the bones, membranes, and muscles; and a further corroboration of this view is found in the pliant condition of the capsular membranes, of the cellular texture, but especially of the two coverings of the spinal marrow, which are beautifully preserved. However, the proof direct consists in Dr. Granville's having been able to separate the wax, by means of combustion and ebullition, from the muscles. He calls the attention of the Society more particularly to the condition of the two nates; one of which was left hard, tanned, contracted, and full of the mummifying ingredients; the other, being deprived of those ingredients, the greatest part of which consisted of bees'-wax, appears like the same part in a recent subject, with the cutaneous pores very distinct, and its muscles, adipose membrane, and vessels, very striking.

The last fact to be noticed, is the presence of several lumps of an earthy matter, mixed with resin, found loose in the abdomen, for the purpose of helping to fill it up, as well as adding to the antiseptic process. Examination proved that the earthy matter was composed of the saline compound noticed on the surface of the body, with argillaceous earth; and, if the embalmers used the water from the Natron lakes, which appears highly probable, it is no less so that they employed also the earthy sediment of that water, and which could be procured in abundance at the margin of those lakes.

The nature of the resin and bitumen employed in the process of embalming, seems not to be a matter of so much interest in the opinion of our author. Some pieces of myrrh were found in a loose state in the mummy here described; but the great art seems to have consisted in impregnating the body with bees'-wax.

From the above details, Dr. Granville proceeds to enumerate the progressive stages by which the Egyptians succeeded in preparing their most perfect, or (as our author calls them) the *primitive* specimens of the art of embalming.

1st. Immediately after death, the embalmers, in the majority of cases, proceeded to remove the viscera, either wholly or partially, sometimes through an incision on one side of the abdomen, in others through the anus. The cavity of the thorax was not usually disturbed.

2d. The head was emptied, either through the nostrils or through one of the orbits; the eyes being taken out, and artificial ones afterwards substituted. The cavity was then washed out by the injection of some fluid, which brought away every vestige even of the membranes, and yet the tentorium still remains. After this, a small quantity of liquid resin was injected into the cranium.

3d. The body was then covered with quick lime for a few hours, and afterwards rubbed with a blunt knife, so as to effectually remove the cuticle; but the scalp was not touched, and the nails were preserved.

4. This having been accomplished, the body was immersed in a liquified mixture of wax and resin, with some sort of bituminous substance; which last, however, does not appear to have been essential to the process. In this preparation the body remained a certain number of days, over a gentle fire; and upon this part of the process the perfection of the mummy appears to have consisted.

5. When the body was taken out of this mixture, it must, of necessity, have been in a very soft and pliant condition. The next step then was the tanning of the surface, and exposing it to the additional influence of the salts, the presence of which has been shown.

Whether the vegetable astringent infusion was a separate process, and the immersion of the body into the water of the Natron lake followed, or the whole constituted one process only, are questions not possible to decide. However, when the embalmers considered that the body was sufficiently impregnated with the active principles employed, it was allowed to dry for a few hours; and then the bandages, previously prepared with tannin, were applied, beginning with each limb; and, whilst this operation was performing, the body must have been in a very supple state, or the deep wrinkles observed on the abdomen, &c. could not have existed. Where it was necessary to obviate the possibility of slackness in some of the turns of the bandages, they were daubed over with two different substances, either wax and resin or resin alone: the lumps of myrrh, &c. were pushed up through the anus.

Such are the principal facts relative to the subject of Egyptian mummies. Some remarks upon the description of this process, as given by Herodotus, next follow, but which we omit, for want of space; merely remarking, that our author ingeniously explains the word mummy to be derived from the Coptic word *mum*, signifying wax: thus strengthening his views by the very etymology of the term.

It only remains to observe, that, by following the processes here detailed, Dr. Granville has succeeded in imitating the preparation of mummies. In one instance a still-born child was employed: this modern mummy has been in existence upwards of three years, without bandage or covering of any kind, exposed to all kinds of temperature, without betraying the slightest vestige of decay or putrefaction.

Our readers may easily conceive the enthusiasm with which our author describes the examination of this perfect specimen, which had probably been prepared upwards of three thousand years; an enthusiasm which the reader of his very interesting essay cannot fail of participating. We ought to have mentioned that the paper is illustrated by several plates.

---

Art. II.—*The Study of Medicine.* Second Edition. By John Mason Good, M.D. F.R.S. F.R.S.L. Mem. Am. Phil. Soc. and F.L.S. of Philadelphia. In five Volumes.—London: Baldwin, Cradock, and Joy, 1825.

We may, perhaps, be censured by some of our readers for not having taken an earlier opportunity of introducing to their notice the above-named elaborate and learned work, which upon the first perusal, we felt convinced would supersede all the Systems of Medicine hitherto in use; an opinion which the lapse of a very few fleeting months has strengthened by the appearance of a second and much enlarged edition. We, therefore, hasten to devote a few pages of our Journal to the consideration of the "Study of Medicine." In so doing, we are well persuaded that we are only performing an act of justice to our readers as well as to ourselves, since we do not imagine that the well-earned celebrity Dr. Good has obtained in the literary and professional world can be in any way augmented by our feeble praise.

It is obvious that a work in five thick octavo volumes, comprising the whole circle of medical science, cannot be susceptible of analysis in the limited space which a monthly publication is enabled to devote to the task: our views must be more humble, —we must be content to give a general view of the nosological arrangement adopted by our author; to point out some of the principal articles most fully or most ably treated of; to mention the additions and improvements which appear in this second edition; and, finally, to recommend its perusal to the profession generally. Without this work, no medical library can be said to be complete; and there is no medical man who need be ashamed of confessing that he has consulted it.

In estimating the ability with which any work is executed, it is necessary to bear in mind the aim proposed by the author; and, though to read a Preface may be "a dull duty," (as has been said of the labour of an editor generally,) it is still a most necessary one, and which, if uniformly complied with, would save many a criticism; since no man is obnoxious to censure for not executing what he did not attempt. Our author's design is, as he tells us, to unite the different branches of medical science, which have hitherto been treated of separately, into a general system, so that the whole may be contemplated under a single view, and pursued under a common study; these branches being physiology, pathology, nosology, and therapeutics.

In speaking of nosology, we cannot but lament that it has become lately the fashion to deride and contemn this branch of medical study; and, because no system has appeared entirely free from objections, and no general definitions have been invented which may not be found to afford occasional exceptions, much undeserved ridicule has been attempted to be thrown upon the arrangements of systematic writers. We are aware that they are all imperfect; that the best of them (until the appearance of that of our author), that of Dr. CULLEN, abounds with faults and imperfections; yet we maintain that a knowledge of his system of nosology will do much to facilitate the study of medicine: it is most useful in enabling us to generalise and to arrange our ideas, presenting that under one view which otherwise would be scattered, confounded, or perhaps overlooked. Some of the defects of this arrangement are pointed out by Dr. Good.

"If it be convenient to concentrate the diseases of the nervous department into one division, as has been attempted by many nosologists, and ably accomplished by Dr. Cullen, it is to be lamented that the same principle has not been allowed to pervade the whole of the nosological plan; and that the diseases of the other chief departments of the animal frame have not been concentrated in the same way, instead of being scattered, as we too often find them, over different divisions of a classification that is itself perpetually shifting from one ground of arrangement to another: which, in one division, as in the Synopsis of Dr. Cullen, by far the best of his day, is derived from the temperature of the body; in a second, from its anatomical structure; in a third, from its chemical depravities; and in a fourth, from its topography: thus offering us in each division a new principle, and one that has no common clue, or analogy with the rest." (*Preface,* p. xii. xiii.)

It was with the hope of obtaining a clearer and more connected method, that our author was induced to frame his system of nosology, which we do not hesitate to pronounce to be far superior to any hitherto in use, and which we do not doubt will soon be universally received in the schools. We do not mean to assert that it is perfect; but we approve of its principle, though it is occasionally carried, perhaps, a little too far, and subdivided and refined upon somewhat unnecessarily. The novelty of names, and their being in most instances derived from the Greek, has with many operated as an objection to our author's nosology; but such objections are merely captious: there is no system that abounds with these names more than that of Dr. Cullen,—his fourth class especially. The poverty of modern language obliges us to have recourse to the inexhaustible riches and the rare flexibility of the Greek tongue; and this remark, whenever made, rather proves the defect of the caviller than of the author of the system.

Of the physiological and pathological portions of this work we have only to say, that the promises which our author makes in his Preface are not broken; and, in therapeutics, we are led to hope that he contemplates an arrangement of medicinal substances upon the same system as his nosology; an arrangement which this branch of the art will admit of, and which cannot fail to facilitate its study, and thereby extend its utility. In therapeutics, Dr. Good has introduced several substances not much employed in this country, or which have fallen into disuse; and he appears to think that, in retrenching the many superfluous articles of the Pharmacopœia of our ancestors, we have, perhaps, gone somewhat too far. He justly remarks, that the substitution of one medicine for another does not put us in possession of an integral representative for that medicine: it may possess the general, but not the peculiar, qualities of the original article.

The Preface concludes with a liberal acknowledgment of the assistance our author has derived from many distinguished members of the profession.

We now present our readers with an outline of the nosological arrangement of Dr. Good. He divides diseases into six classes —Cœliaca, Pneumatica, Hæmatica, Neurotica, Genetica, and Eccritica; the last being the diseases of the excernent function, —the others are too obvious to need explanation.

The first class has only two orders: the first is Enterica, or diseases affecting the intestinal canal; and this order includes all diseases of this function from the mouth to the anus. The second order is Splanchnica, or diseases of the collatitious viscera.

The second class has also two orders: the first Phonica, or diseases affecting the vocal avenues; and second, Pneumonica, affecting the lungs, their membranes, or motive power.

The third class, Hæmatica, includes four orders: namely, Pyretica, or fevers; Phlogotica, or inflammations; Exanthematica, or eruptive diseases; and Dysthetica, or Cachexies.

The fourth class, Neurotica, or diseases affecting the nervous function, is equally extensive; it comprises also four orders: Phrenica, affecting the intellect; Æsthetica, affecting the sensation; Cinetica, affecting the muscles; Systatica, affecting all the sensorial powers simultaneously.

Class the fifth is termed Genetica, and has three orders: Cenotica, affecting the fluids; Orgastica, affecting the orgasm; and Carpotica, affecting the impregnation.

The sixth and last class, Eccritica, includes three orders also: they are Mesotica, affecting the parenchyma; Catotica, affecting internal surfaces; and Acrotica, affecting the external surface.

We cannot, of course, pretend to enter into the minute subdivisions of this arrangement. In thus enumerating its principal features, we have given such of our readers as have not had an opportunity of seeing Dr. Good's work, some idea of its general character, and much food for reflection.

We shall now proceed to notice the additions and alterations which this second edition presents. Under the last head will be found such additional information on the subject of spasmodic cholera, of dysentery, or yellow fever, and some other diseases, as the progress of medical knowledge in the short space of time that has elapsed since the first appearance of this work had enabled the author to collect. His observations on plague will also be found highly worthy of perusal; and he has devoted much space, and paid great attention to the modern doctrines and writings on those forms of disease resembling syphilis. In this inquiry, although we differ entirely from the learned author, we must yet allow that he has shown his usual industry in the collection of his materials, and no less skill in arranging them.

Of new matter there is so great a quantity, as to account for the extension of the work to a fifth volume: the most important subjects treated of are, the fevers attending on dissection with a punctured or abraded hand, the melanosis, lateral distortion of the spine, the mollities cerebri, and a peculiar species of trichosis, besides some few others.

Dr. Good, in replying to the criticisms and objections that have from time to time appeared against certain parts of his work, evinces a spirit of moderation and candour, strongly contrasted with the petulance displayed by many of the writers of ephemeral productions; whom to contradict or to doubt, is at once to convert into irreconcileable enemies. Our author, on the contrary, attends to many of the hints that have been given to him; and, where he still retains his own opinions, he enforces them with reasons that are, in most instances, satisfactory. The only point of importance, he says, which he has found a difficulty in acceding to, is that of introducing generally a description of the appearances offered by diseases on dissection, excepting where they are strictly pathognomic. The following reasons are urged by Dr. Good for this omission:—

"First, because the present is not designed to be a sepulcretum, or work on morbid anatomy; and would have been swelled to nearly double its extent, if such a connexion had been allowed. And, next, because, however valuable an expert practice in dissection may be to a student in the field of anatomy, in a pathological point of view its developments, except where strictly applicable and illustrative, will more frequently perplex than instruct him. They will rarely give him any information concerning the elementary or chemical changes that have taken place in the animal fluids; and may lead him, in a thousand instances, to mistake effects for causes,—the result of symptoms or accidents for that of idiopathy, even in morbid changes of structure. The truth is, as M. Foderé has justly observed, that by far the greater number of diseases are the products of disordered vitality before they become organic: and when at length they assume such a character, it is as a consequence rather than as a first moving power." (*Advertisement*, p. ix. x.)

We are of opinion that there is much truth in these remarks of M. Foderé, and yet we by no means intend to discredit *generally* the benefit derived from the dissection of diseased bodies; but it is only from the mass of information thus obtained, that any real advantage is derived: from any single examination, we should be very cautious as to what conclusions we deduce. We have notable instances every day afforded us by the continental physicians, how common it is to see a very minutely-detailed morbid appearance following the history of a disease treated in the most puerile and trifling manner: indeed, it would seem, in many instances, that the physician's only hope and expectation was to give a learned account of the patient's dissection, as if that was the ultimate end and object of his attendance. As truth it very frequently happens so. We are thus satisfied, that the patient died perfectly cured.

### DIVISION II.

### FOREIGN.

ART. III.—*A Compendious System of Midwifery, chiefly designed to facilitate the Inquiries of those who may be pursuing this Branch of Study. Illustrated by occasional Cases; with fourteen Engravings.* By W. P. DEWEES, M.D. Lecturer on Midwifery, Member of the American Phil. Soc. &c.—8vo. pp. 628. Miller, London, 1825.

[Concluded from page 81.]

"*Of the Dilatation of the Os Uteri.*"—DR. DEWEES ente[illegible]ains some peculiar notions upon the causes of this phenom[illegible]on, which he states much in detail. The manner in which he conducts the inquiry evinces a patient and laudable determination to investigate minutely every part of so important a subject, and not to pass to conclusions upon the unproved authority of other writers, however high their professional rank. No solution has ever yet been given, we believe, to the question of "what is it that gives the uterine contractions the alternate or periodical form?"

"If we fail," says Dr. Dewees, "to give a just one, it must be remembered we but hazard a conjecture: if it fail in probability, it will but share the fate of the thousands of others that have been given, from the time of Hippocrates to the present moment; and we most honestly declare, we have no overweening tenacity upon this or any other hypothetical subject, and will most cordially adopt any other that shall appear to possess higher claims for either ingenuity or truth.

"In order that a muscle shall renew its contraction, it must by some antagonising power be elongated, after it has become relaxed: in almost every part of the body this power is at once discoverable; but where, and in what, does that reside which enables the uterus to repeat its efforts? We are of opinion this power resides within its own structure and economy: we shall now attempt to prove this. The uterus, by impregnation, becomes of course distended, in proportion as that process itself advances: it is, therefore, elongated, or its fibres put to a certain extent upon the stretch, and are thus ready to contract so soon as the appropriate stimulus is applied. What is the effect of this contraction? An approximation of the uterine fibres; a compression of all its blood-vessels, with the immediate discharge of a large portion of blood from them into the general system: in consequence of this, the uterus becomes paler, and the vessels empty, or nearly so. The blood escapes, by means of this contraction, *quâquâ versum;* and, to facilitate its departure, the anastomoses between the arteries and veins are unusually frequent; and the latter are not furnished with valves.

"What is the effect of the subsequent relaxation? The fibres of the uterus become longer, straighter, and more easily distensible; the large vessels and sinuses are less compressed, and consequently will not permit the natural resiliency of their coats to act; while the influent blood will suddenly fill them, and thus restore the equilibrium which the previous contraction had destroyed. Now, this rapid influx will not only distend the emptied vessels, but will also prove a powerful stimulus to the uterine fibres, and thus enable them to renew the contraction; and this will be repeated from time to time, until there is no further necessity for its continuance. This plethoric state of the uterus, if we may so term it, is proved by the heightened colour of its parietes." (P. 177, 178.)

In his observations upon the manner in which the os uteri becomes opened, the author observes, that, "during gestation, at least until the seventh month, the longitudinal fibres yield much more willingly to the distracting force of the increasing ovum, than the circular: this may be owing to their greater length or their greater laxity, and hence, perhaps, the lengthened form of the uterus. They must have, however, their maximum of stretching; and, when this period arrives, they will be stimulated to contraction."

Upon a subject of speculation the field lies open, and we would reply to this paragraph by offering a doubt as to the accuracy of the hypothesis it contains. If a muscular part is suddenly extended, its contractile powers are brought into violent action; but let the part be extended gradually, as is, of course, the case where the extension depends upon the slowly increasing size of the ovum, and when it has arrived at its "maximum of stretching," its antagonistic powers appear to be destroyed; for contraction either does not take place at all, or very imperfectly. It is well known that surgeons act upon this principle, when they have to overcome the obstinate contraction of muscles: they exhaust the powers of the part by keeping up a gradual extension.

On the *Conduct during Labour*, many sensible suggestions are thrown out.

"When all things are doing well, the duties of the accoucheur are limited indeed: it is but where the contrary obtain that he can be said to be actively useful; but, to discriminate between these two conditions, requires a thorough knowledge of what a healthy labour consists; and this he can only with certainty know by being well grounded in the principles of his profession, aided by an extensive, or at least a well-directed, experience.

"To conduct a labour with safety, the practitioner should be well acquainted with its phenomena; the order, or succession of them; where certain of them may be wanting, or are in excess; the relative or positive importance of such; the force or effect of each pain; the necessity of preserving or of wasting the waters; the resistance the os uteri or external parts may offer; the situation of the former as regards the presenting part; the certainty of the presentation, both generically and specifically; the mode of rectifying any error of presentation in proper time; the capability of doing this with the greatest advantage to the patient and to the infant; and, 'though last not least' in importance, is the pursuing a firm, candid, and feeling conduct throughout the whole scene, that he may not be betrayed into indiscretion, by the overweening anxiety of the friends of the patient; that he may not lose the important moment to act, from the apprehension that blame may attach upon the disclosure of its necessity; and that the poor suffering woman may derive every advantage his kindness and sympathy may afford.

"That man is but little used to the exercise of the social virtues who is ignorant of the influence a kind and feeling conduct has upon his suffering patient: to her, it almost atones for the want of skill or experience; and, to deprive her of it, is withholding a right, for which nothing beside can compensate.

"She is entitled to all the consolation a well-grounded assurance of a happy termination of her sufferings can afford; and this should not be withheld from time to time, that she may profit by its encouraging influence; yet she must not be betrayed into false hopes, by an ill-judged promise of a speedy issue, when the period, from the very nature of the case, must be very remote. Nothing, perhaps, is so destructive to confidence as the ill-requited promises of this kind; nothing so sickening to the heart as this 'hope deferred.'

"The young practitioner should be very sparing of promises; for it requires long experience to make them with any kind of certainty; and until then they should be evasively given, that sad disappointment may not ensue: for a woman will support herself with much firmness, where relief is believed to be even very distant, but certain; while she would flag under the failure of often-repeated promises of speedy relief. Her mind should be kept as free from anxiety as the nature of her situation would permit; therefore, no conversation should be indulged in that might for an instant excite her apprehensions: conversation should be cheerful, and free from the idle discussions of danger from similar situations, and should be as void of levity as of gloom." (P. 185—7.)

A more mischievous error cannot be committed in the management of labours, than the *improper* rupture of the membranes. In several instances we have known the life of the patient placed in jeopardy, by the ignorant officiousness of midwives, who have ruptured the membranes in cases of presentation of the arm, and have afterwards waited perhaps forty or fifty hours before they sought our assistance. In three cases of this sort, which have not long ago occurred, we have known these meddling matrons accost the practitioner by the consolatory assurance, "that every thing was complete, and as dry as a bone." Upon examination, a hand has been found in the vagina, and the uterus powerfully contracted. He who has performed the operation of turning under such untoward circumstances, can alone estimate the injury inflicted, and the melancholy increase of suffering which such criminal interference gives rise to.

Having premised the necessity of caution, we give the following extract:—

"Should the pains be efficient, and the os uteri well dilated, or even easily dilatable, and the membranes entire, let them be ruptured by the pressure of the finger against them, or by cutting them by the nail of the introduced finger; and this should be done for the following reasons:—first, because, when the mouth of the uterus is dilated, or even easily dilatable, the membranes have performed every duty they can perform; secondly, that very often the advancement of the presenting part is retarded by the strength of the membranes, and the labour much protracted by it; thirdly, that very frequently the pains are increased, both in force and frequency, and the labour much abridged by it; fourthly, it gives much greater security to the woman after delivery, by permitting the tonic contraction to take place before it is accomplished, and thus ensuring a more speedy delivery of the placenta, and a greater chance of exemption from hemorrhage." (P. 189, 190.)

In the next section, *the proper management of the child* is briefly discussed. We are recommended to apply but one ligature upon the funis, except in the case of twins. No harm can possibly arise from the application of two ligatures, and we consider it the safer practice. Before the delivery of the placenta, the hand of the practitioner should always be laid upon the abdomen, to determine the state of the uterus, and also whether there still remains another child. If this simple and necessary precaution had been always adopted, many blunders would have been prevented.

"This examination will discover the uterus in one of two conditions, —namely, contracted or uncontracted. If the first, the placenta may be delivered, provided it be loose in the vagina, by tightening the cord with the left hand, and tracing it with the fore-finger of the right to the placenta, which we hook with the finger introduced, and gently draw by the cord with the other hand, until it pass through the os externum; we should then grasp it with both hands, and give it several twirls, to twist the membranes, that they may be withdrawn from the uterus whole. The advantage of this is, we prevent a terrible stench, by thus removing them; and, second, we prevent great alarm sometimes: we have known them about to escape from the vagina a few days after delivery, to the great terror of the patient and her friends, by being mistaken for the uterus falling down.

"When the placenta is delivered, it is to be carefully placed in a bason or pot, and then removed. The abdomen should again be examined, to ascertain the condition of the uterus: should it be well contracted, which is easily determined by its hardness and size, we should entertain every reasonable hope that every thing is so far well; but, should the uterus be found flaccid, brisk frictions with the open hand, with the occasional grasping pressure of the fingers, should be instantly instituted. Should these be successful, the uterus will be found to harden gradually, as well as to diminish in size under the hand. At this moment, perhaps, there may be a sudden discharge of coagula from the vagina, accompanied by some pain, which very frequently gives great alarm to the inexperienced practitioner, and renders him doubtful of the propriety of the plan he is pursuing; but, so far from being alarmed at this circumstance, he should felicitate himself upon it, as it is a proof the uterus is contracting. The frictions should, however, be continued for some time, or until the uterus becomes very hard, and appears to be fast retiring within the pelvic cavity." (P. 194, 195.)

*Of After-Pains.*—" There is a remarkable circumstance attending these pains which deserves notice, if it cannot be explained; which is the almost uniform renewal of them, upon the application of the child to the breast, if they have been suspended even for hours; and the aggravation of them, if they have not been controlled." (P. 197.)

Dr. D. recommends ten-grain doses of camphor for the relief of after-pains. We should prefer a union of hyoscyamus with purgatives. Opium may be necessary. Whenever the after-pains are severe and obstinate, our suspicion should be excited by any febrile movement in the system. Dr. Dewees has met with a few cases of a very distressing kind, which he is not aware have attracted the notice of any previous writer.

" It is a most severe and constant pain at the very extremity of the sacrum and coccyx; it begins the instant the child is born; and perseveres, with most agonising severity, until its violence is overcome by the rapid and liberal use of camphor and opium. It is declared to be, by the patient, infinitely more insupportable than any pains of labour; for it is never ceasing.

" The first case of this kind we met with occasioned us no little anxiety and perplexity, from both its novelty and severity. It was with a young lady with her first child. It began most ferociously the instant the child was born, and its severity was such as to make us abandon the delivery of the after-birth, to attempt relief to our sorely-afflicted patient. We at first looked upon it as only a protracted after-pain, which we did not expect to encounter with a first child. We immediately gave a large dose of laudanum, and repeated it in fifteen minutes: at the end of a quarter of an hour, as there was no abatement of the suffering, we again gave the laudanum. This procured no relief in half an hour more, though, in the three doses, more than two hundred drops of this medicine were given in the course of three-quarters of an hour. We were obliged now to suspend the repetition of the laudanum, from a fear of an excess in its exhibition; but, to amuse the patient, we gave her a few drops at a time, disguised by a little of the compound spirit of lavender, until an hour had passed. By this time the patient thought herself a little easier, but still suffering very severely. We now ventured upon another full dose of laudanum, and sat down to deliver the placenta: this was quickly done, as it was found lying loose in the vagina, but its expulsion procured no alteration in her sufferings. In a word, nearly five hundred drops of laudanum were administered before complete relief was obtained. After she became easy, she had no subsequent return of pain of any kind, nor did she suffer in the least from the liberal use we made of laudanum in so short a period of time.

"On the termination of her next labour, as she had most anxiously and fearfully anticipated, the same violent distress assailed her. We instantly gave her at one dose 120 drops of laudanum, which was repeated in twenty minutes: in the mean time, the placenta was spontaneously discharged. This second dose afforded no relief, and we were induced then to administer the laudanum at short intervals, which, as before, eventually overcame the pain. As on the former occasion, she suffered nothing after this pain was subdued.

"On her third confinement, we were again distressed to find a recurrence of this terrible agony: we had, however, from our former experience in her case, anticipated this event, and had at hand the following julep:—

℞. Gum. Camph. ʒij.

Sp. Vin. rect. q. s. f. pulv.—Add

Pulv. G. Arab. ʒiij.

Tinct. Opii acetat. ʒijss.

Ol. Juniperi, gut.xx.

Sacch. alb. q. s.

Aq. font. ℥vj. M.

Of this a large table-spoonful was given, about fifteen minutes before we expected the child to be born, by way of making some impression before the pain should come on. The child was born rather within the period we had calculated, and, as on the two former occasions, the pain commenced the instant it was in the world. Another spoonful of the julep was immediately given, and this was followed by another in fifteen minutes more, which now had decidedly abated the severity of the pain; and we had the satisfaction of seeing it entirely conquered in an hour from its commencement,—a period of less than half the length of the former paroxysms. The placenta came away without any trouble, as it had always done before. It was, we thought, evident that the combination of camphor and opium was highly beneficial; and perhaps they were aided by the oil of juniper, which we had frequently found highly useful in controlling after-pains. On her fourth delivery she was managed precisely as above related, and with the same happy effects." (P. 198—200.)

The degree of severity of the after-pains will depend very much upon the conduct of the practitioner during labour. We may render them less severe, or perhaps even prevent their occurrence—

"1st. By rupturing the membranes whenever the mouth of the uterus is sufficiently well dilated to permit the head to pass, that the tonic contraction may immediately ensue: by this the following advantages result, as regards the prevention of after pains. By the absence of the waters, the uterus is reduced in size in proportion to the quantity discharged: this gives greater strength to this organ, and enables it to contract with more force when empty, and consequently will more certainly diminish the size of the vessels exposed by the separation of the placenta, which, pouring out blood, gives rise to these pains. Again, it prevents the uterus from being too suddenly emptied, and thus inducing a state of atony in it; for it must be remembered, that after-pains are never more certain, nor ever more severe, than after a very quick labour. 2d. By permitting the uterus alone to finish the labour after the head is born: in doing this, we have an assurance that the tonic contraction has regularly followed, as the uterus became more and more empty; for, were this not the case, the alternate contractions would be feeble and transitory, as always happens when the shoulders are hurried through the external parts, and the uterus too suddenly emptied. The tonic contraction, of course, in this case, is imperfect; and consequently the vessels are not pressed upon by this power, when exposed by the separation and departure of the placenta,—consequently, blood is freely poured into the cavity of the uterus, where it coagulates, and obliges the uterus to throw it off by repeated contractions. 3d. After the delivery of the child, we may do much, by not attempting the delivery of the placenta until we have insured the tonic contraction of the uterus, by the frictions before recommended over the hypogastric region; and, after its expulsion, to repeat them, until the uterus seems to retire considerably within the pelvic cavity. Burton's success (though we should be but little disposed to follow his practice,) in preventing after-pains, by the introduction of his hand to the fundus of the uterus, and there kept until he found this organ contracting upon it, depended entirely upon the principle we have been endeavouring to establish,—namely, promoting, as quickly and as certainly as possible, the tonic contraction of the uterus." (P. 201, 202.)

In cases of suppression of urine after delivery, the use of the catheter "must be repeated until the bladder regains its powers, and is capable of discharging its contents; it sometimes becomes necessary to do this twice or thrice a-day, but in general once will do." If it were our only purpose in such cases to prevent the woman from suffering pain from the suppression of urine, it is true that the use of the catheter once a-day "will generally do," (to adopt the phraseology of our author.) But we have a much more important object in view,—to prevent the natural power of the bladder from being so far weakened by over-distention, that a very considerable time may elapse before the patient is able to pass her urine. We have known the use of the catheter necessary for many weeks, in consequence of the contractile power of the bladder having been lost, from the water not having at first been drawn off more than once in twenty-four hours. We differ, then, from our author upon this point. The introduction of the catheter once a-day ought never to be considered sufficient: it should be employed at least twice in the day; and it is probable, if it were used still more frequently, that the bladder would have a better chance of speedily recovering its power.

In the treatment of the *menorrhagia lochialis*, after having premised bleeding and purging, we are told "we may give from a grain and a half to two grains of the acetate of lead, every three or four hours." We have generally found that such cases were relieved by keeping the patient cool, administering acids, the use of astringent injections, and frequently dashing cold water upon the pubis and back.

We must pass over the brief consideration of the several diseases to which the new-born infant is liable, with but one remark. The introduction of the catheter is spoken of in the case of a child ten days old. We have never had occasion to employ the instrument at so early an age, and fear that its employment is more easily described on paper than carried into execution in practice, particularly in the male. The following case is not unworthy of a moment's consideration.

"We will relate," says our author, "a curious instance of the influence of an aching tooth upon the secretion of milk, and its indirect agency in producing the 'belly-ache' of the first form. Mrs. ——— was delivered of a fine healthy-looking boy, which appeared to do perfectly well for the first two weeks after birth: at this time it became uneasy, and frequently cried. The usual domestic remedies were employed from time to time for its relief, without the smallest benefit: the complaint seemed to increase every day; the pain was more severe, and longer continued; the stomach and bowels became affected, the one with sour vomitings, the other by frequently discharging green stools. The child could obtain no relief but from laudanum, and this we were obliged to give in large and constantly increasing doses. The emaciation was so great, as to render the child lighter at three months than when first born. In this situation did things continue, without much aggravation or amendment, until the child was five months old. By this time it was (without a figure) nothing but skin and bones.

"At one of our visits, we observed the mother apply her hand very suddenly to her face, and press it forcibly, as if in pain from a tooth: we inquired of her what she ailed? she informed us she was very much tormented, both by day and by night, by tooth-ache, and had been for some time before the child was born, and ever since. We immediately declared our opinion that this was the cause of the affliction of her child; the constant pain she was enduring, and the great loss of sleep, so affected her stomach, and indirectly the breasts, that they could not yield a healthy nourishment; and advised her immediately to send for a dentist, and have the tooth extracted. This was accordingly done; and from that day the child began to recover, and in a short time was perfectly restored to health." (P. 229, 230.)

In a work principally designed for the use of students, the recommendation for giving "large and constantly increasing doses of laudanum" to a child of three weeks old, should be much more definite. If we oppose "sour vomitings and green stools" by the long-continued use of laudanum, we must expect to have "the emaciation great," "the child lighter," and in some time nothing "but skin and bones." From the imperfect relation which is presented of this case, we are most decidedly of opinion that the practice employed was highly injudicious. We doubt also the relationship between the tooth-ache of the mother and the belly-ache of the child. We earnestly entreat the young practitioner to proceed with the utmost caution in the use of even very minute doses of laudanum to very young children.* In treating infants, it should be remembered that the decrease of a dose of a particular remedy according to the common scale, having assumed a given quantity to be a proper dose for the adult, is not sufficient. For example, calomel may generally be administered in a much larger dose than would be laid down as proportionate to the difference of age: the reverse is the case with opium, if, indeed, it be possible to give any abstract and arbitrary rules for the exhibition of a medicine, the effects of which are so much under the control of individual idiosyncrasies.

We now enter upon the process of *Labour.* Six varieties of head-presentation are admitted, in compliance with the arrangement of BAUDELOCQUE, of whom our author is a warm admirer. It would be easy to multiply these presentations, but we should gain no practical advantages by so doing. Plates are given at the end of the volume, which are explanatory of the mechanism of each head-presentation: they should be carefully studied.

*Preternatural Labours.*—There are many causes which may convert a natural labour into a preternatural one. "A labour may commence with the most favourable appearances of being as speedily as successfully terminated; but, after a continuance for a longer or a shorter time of the fairest promise, the patient may be assailed by some accident, which puts in jeopardy her life or that of the child, or both, and from which nothing can save them but the well-directed and timely interference of art." (P. 249.)

*Flooding* and *convulsions,* two of the most formidable concomitants of labour, are treated of in subsequent chapters.

*Syncopes.*—In some women, fainting occurs from any extraordinary excitement, or even slight pain.

"But when these faintings take place where this peculiarity of constitution will not account for them,—where they are attended with increasing exhaustion,—where the labour-pains diminish, both in force and frequency,—where they become more permanent in their duration, and the pulse flags or becomes nearly extinct,—it behoves the practitioner to discover, when practicable, with all possible speed, the cause, and as quickly as may be to remove it.

* See a case in which an infant, ten days old, was poisoned by a tea-spoonful of syrup of poppies, in the Number of this Journal for March 1823.

"An internal hemorrhage is perhaps the most frequent cause of this alarming condition. When it proceeds from this source, it always commences gradually,—that is, the debility is not suddenly induced, nor are the syncopes at first profound; but both may pretty rapidly increase, in proportion to the extent or force of the remote cause. The abdomen is observed to enlarge; sometimes there is a slight external hemorrhage, or discharge of serum a little tinged with blood; the pains slacken, and the woman becomes exhausted.

"In cases like these, there appears to be but one remedy, which is immediate delivery by turning, provided the uterus be in the condition, already sufficiently often indicated, to permit this operation; and, if not, we are pretty certain there is not that imperious necessity for instant delivery that would put to defiance the rules we have endeavoured to inculcate against a forcible entry of the uterus for any purpose; for it must be recollected that, after labour has commenced, and made some little progress, and especially if the woman has gone to the full period of utero-gestation, that the disposition to syncope is oftentimes favourable to the dilatation of the os uteri, or, at least, renders it so pliant as to be penetrated with but little force. When this is so, turning is the remedy; but we must take care to secure the tonic contraction of the uterus before we attempt the delivery of the placenta.

"Baudelocque relates cases of concealed hemorrhage, which are highly interesting and well worth consulting. From what he relates upon this subject, it would appear that an hemorrhage of this kind may take place long before, as well as near, the period of nine months, and that the immense distention the uterus suffers from the influent blood provokes it to contraction, and brings on labour-pains. But, as the cause which may produce indicative syncopes cannot always be ascertained, and as it is rational to suppose it is in some way or other connected with labour, it will be well, under proper conditions of the uterus, to turn, and thus remove a difficulty, if not the absolute or direct cause of the faintings. Should these occur when labour is far advanced, or when turning would be ineligible, the forceps must be used." (P. 252, 253.)

Fainting fits strongly resemble hysteria, and have probably been sometimes mistaken for true puerperal convulsions. We must observe, that, although a female may be constitutionally subject to fainting, it is a symptom which is never to be regarded with indifference during labour, although it may frequently not demand our active interference.

An unfortunate and fatal case is recorded by Dr. Merriman, in which the patient fainted during labour. She quickly recovered, and no notice was taken of it. On the third day after delivery, during the operation of an aperient, she suddenly expired. This patient was not under the care of Dr. Merriman.

*Partial Contractions of the Uterus.*—

"By these we are to understand the contractions of the external edge of the mouth of the uterus, as well as that portion which, in the unimpregnated state, constitutes the internal edge or orifice of this organ, around the neck of the child, so as to prevent the descent of the shoulders. The first of these conditions is the most serious in its consequences, because most difficult to remedy. In this case, the head of the child has escaped through the external ring, constituting the mouth of the uterus; in consequence of which, this part retracts itself behind it, and then, no longer being upon the stretch by the bulk of the head, it contracts, and this so strictly sometimes as to embrace the neck. When this takes place, the bulky shoulders cannot pass the barrier which the contracted neck offers, and they are therefore immediately arrested, and their form is but ill calculated to dilate again the mouth of the uterus; for now it can only be opened by mechanical means.

"In the second case, the head remains enveloped in the lower portion of the uterus, (which portion, in the unimpregnated state, constitutes the neck,) while the internal edge contracts, but not so strictly, round the neck, and thus offers on all sides an inclined plane for the shoulders to rest upon. This contraction is much more frequent than the former, and is decidedly the greatest obstacle we have to encounter sometimes, when we attempt to turn after the waters have long been drained off. It will readily be perceived, that it is essential to either of these cases that the waters are discharged; and, as far as our own experience will justify the remark, that neither of these contractions take place, but after the lapse of a considerable time, at least to the degree that would seriously obstruct delivery.

"These cases necessarily result from the constant disposition of the uterus to return to its original size, after the distracting cause is removed; and this, as we have elsewhere observed, is by virtue of the tonic contraction of the uterus, and its constant tendency to accommodate this organ to the shape and inequalities of its contents: hence the contractions in question.

"When either of these conditions complicate the labour, it will become stationary, or nearly so, for many hours; and, whatever other cause may combine with the existing one to render immediate delivery desirable or indispensable, it will be found almost impracticable to perform it by almost any means. If we attempt to turn, we shall find it almost impossible to insinuate the hand into the orifice of the uterus, to dilate it sufficiently to permit it to pass to the feet; and, if we apply the forceps, we can only deliver at the risk of tearing the uterus, especially in the first of these cases. In the second, Baudelocque says, 'though it may in some cases produce as great an obstacle to delivery, it is always easier to overcome it, and the same inconveniences do not result from it; because the head is not so far engaged, and may always be pushed back, which permits us to advance the hand under the uterine circle in question, and dilate it.' We do not altogether agree with this high authority on this point; for we have certainly met with this case, where we could not push back the head, and thus dilate the stricture; and also we have found there was no possible advantage in merely overcoming this resistance by passing the hand pretty forcibly through the contraction, so long as the stricture continued at all in force, after the hand was thus passed. For, if the contraction be not entirely removed, or so weakened as to yield to a moderate force, there is nothing gained by bringing down the feet to the orifice of the uterus, or even lower; for, the instant the breech descends to this stricture, its further progress is arrested by the inclined plane we have just spoken of, and no force that could safely be exerted will make it pass through this narrowing of the uterus." (P. 256–258.)

The first case here described is rare; the second is very common. In ordinary practice, they pass under the convenient and undefined term of "a lingering case," the practitioner not having a clear notion of the cause of the delay. Bleeding largely in such cases is strongly recommended. This practice is much more common in America than in this country, and it is carried to a greater extent. We look back with regret to some cases of long procrastinated labour from rigidity of the uterus, which formerly occurred in our own practice, and in which we confess we are apprehensive the result might have been more favourable, if blood-letting had been carried further. As a proof that irregular and partial contractions of the uterus are frequently occasioned by the officious and improper handling of the parts during labour, we may observe, that a *sage femme* of our acquaintance, who is very far removed from a *femme sage*, has frequently called us in to such cases. This woman is notorious for being a "fine helper:" she commences the treatment of every labour by using the roughest endeavours to stretch the parts, and thus almost ensures irregular or ineffectual action of the uterus.

*Cases in which the Face presents.*—Some difficulty, or at least unusual delay, may always be expected in these cases. By some authors, we are recommended always to turn in face presentation; Dr. Dewees does not recommend this practice indiscriminately. In our own practice, we have had many cases of this variety of presentation: the operation of turning we believe to be seldom necessary. Patience and the efforts of nature will mostly terminate the labour, with safety to both mother and child. In this opinion we are supported by many excellent authorities. Dr. Merriman, for example, states that "the management of this case must, in a great measure, be left to nature and time, which will gradually effect the delivery. Dr. M. has known two instances in which the presentation of the face was converted, by the pains alone, into a natural presentation. The inexperienced practitioner is to guard against any expression of surprise at the probably swoln, and perhaps livid, appearance of the face, when it has presented. The eyes may be tumid, and the features entirely distorted. We are not aware of any case in which the natural appearance of the parts has not been regained in the course of a short time; provided, indeed, no improper interference or violence has been inflicted by the ill-directed efforts of an unskilful hand. Such labours are almost always very tedious. We disapprove of the practice of "again and again" attempting to alter the position of the face. We doubt not that Dr. Dewees, and many others of equal skill and experience, would continue these efforts without absolute injury to the patient, although *we* are not "pretty certain of success under a well-directed management," unless the presentation is discovered early. We feel confident that if, in the general course of practice, the manual efforts which are described by Dr. D. in face cases be attempted, that much mischief must accrue to the mother or child, in many instances.

Under the head of *Exhaustion*, some interesting observations will be found.

In the management of *Preternatural Labours*, it is to be remembered that, although the operation of turning may frequently be necessary, and is consequently recommended by every authority, we are not justified in violently attempting to overcome the opposition of a rigid os uteri. Patient and moderately continued efforts are more than equivalent in effect to hasty and rude exertions, and can inflict no injury. We are induced to dwell for a moment upon this subject, because we know that practitioners not unfrequently consider the operation of turning as one which they are called upon to perform, without any reference to the state of the parts. "Our conduct must be regulated almost exclusively, as regards delivery, by the condition of the os uteri." The author might with propriety have added, that, where turning is necessary, and there exists much rigidity of the parts, bleeding may generally be practised with advantage. We may be required to turn in cases of hemorrhage. In such cases, rigidity will seldom oppose our efforts. The accidental occurrence of discharge of blood has produced the same effect that would result from artificial bleeding.

The *position of the woman for turning* varies. Dr. Dewees prefers laying the patient on her back; we have always succeeded very well with the woman upon her side. The arrangement of the bed is of importance, not only for the comfort of the patient, but for the convenience of the practitioner. We have known the accoucheur foiled in his efforts to turn, and obliged to withdraw his hand from the uterus, in consequence of having, from neglect of this precaution, allowed the patient to be so completely buried in the middle of a large feather-bed, that he could not act with freedom. A second attempt was objected to by the friends, and our assistance was required. The patient having been properly placed upon a mattress, the operation was performed with facility. We must refer to the work itself for the detailed rules upon this subject.

One word we may be allowed to offer in reprobation of the disgusting and unnecessary parade which some practitioners invariably make, when they approach the labour-bed. Their coat-sleeves are tucked up as high as possible,—perhaps the coat is taken off; their arms are bared, and an apron called for. In some cases this preparation may be necessary, but it is decidedly improper in the great majority of labours. It is a puerile attempt to convince the by-standers that a formidable duty is to be performed; and it creates much alarm and disgust in the mind of the patient.

The mode of performing the operation of turning is minutely described. It is observed, that "the whole act of turning should be considered as one of necessity rather than of choice; therefore, when it is proper to commence with it, it is, we believe, *always* proper to finish with it, and not trust the delivery to the powers of nature, after having brought the feet into the vagina, as recommended by some." We would substitute the word "generally" for "always." In most cases the uterus will be exhausted by the operation, and it would be to no purpose to rely upon nature: she would not be capable of effecting the delivery. But where the case is favourable, if the parts are well dilated, and but very gentle efforts to turn are sufficient, it will frequently happen that, when we have brought the feet into the vagina, the ordinary action of the uterus will ensue, and our interference will not be necessary.

In the eighteenth chapter, the mode of operating in each particular case of head presentation is laid down.

The subject of the *Forceps* is highly important, and is considered much at length. Dr. Dewees prefers, from "an experience of many years, the long French, or the Baudelocque forceps." He says "there is no situation of the head which can be delivered by the short forceps, that cannot, with at least equal certainty, be relieved by the long; but that there are situations of the head which the long forceps can deliver, where it is entirely impossible to relieve by the short: this, in our estimation, is conclusive." In our own practice we have invariably used the short forceps, and in no instance have we failed in completing the delivery with them. The manner in which the French forceps lock has certainly many advantages. No portion of the soft parts, or hair of the pudendum, can be grasped by them; and they lock without the vulva, even in high situations of the head. Habit, however, will not only reconcile us to a particular instrument, but will also enable us to use it with much more dexterity than one which may be in itself superior, but to which we are not accustomed. That much more force may be employed by the French instrument, must also be granted; and it is for this very reason that we should deprecate its use in common practice. In skilful hands, no danger need, of course, be apprehended.

"Position," says Dr. D. "is a great point in the application of the forceps; but, as regards the particular situation of the woman for delivery, there is a diversity of opinion between the British and continental practitioners; and the same may be said of the different accoucheurs in our own country, depending very much upon the school they have been educated in, or the authority they are the most in the habit of following. The British practitioner almost invariably directs his patient to be placed upon her side, with her hips near the edge of the bed; while the continental accoucheur has her placed upon her back. It is, perhaps, not very difficult to explain the cause of this difference: the British practitioner never, or at least with very few exceptions, since the days of the well-instructed and judicious Smellie, attempts to deliver the head from the superior strait, while many of the continental accoucheurs do: in the first, the lateral position of the woman is, perhaps, as eligible as any; but, in the second, it would be impossible in that position to deliver from the superior strait. Now, as the position of the back enables the practitioner to deliver from any part of the pelvis when necessary, it should always, we think, be preferred; and more especially as the relative situations of the head and pelvis will be better understood by the young practitioner, as he will constantly have the symphysis pubes as a guide, to determine with precision every other part of the pelvis, which he cannot so exactly do when the patient is on her side." (P. 298.)

We are properly instructed always to apprise the friends of the patient, of the necessity of the artificial aid of the forceps. Even the most uninformed and timid may be speedily reconciled to the employment of this instrument, if they are described or shown to be nothing more than an additional pair of hands.

With all our anxiety to enter into the deliberate consideration of the various important topics which the volume of Dr. Dewees embraces, we are compelled, from the space we have already occupied, to become brief. We must comment only upon those points which are discrepant from the views which are recognised amongst ourselves, and which are therefore particularly interesting.

Dr. Dewees has, in another publication, commented upon the aphorisms of Dr. Denman, which are so celebrated in this country. He is, however, upon the present occasion, not sparing of censure upon the subject of Dr. Denman's opinions respecting the use of the forceps. We would have every difference of opinion stated without the least appearance of acrimony. The ardent wish of the late highly respected Dr. Denman, and that of our author, has doubtless been to present, to the best of their abilities, the results of their own experience, and a summary of the received practice of their respective day. Progressive improvements have shown that Dr. Denman confined the use of the forceps within too narrow limits. His humanity, perhaps, induced him to pause in the recommendation of more frequent instrumental assistance, from the apprehension that the ignorant might be inspired by boldness, and that positive injury would be inflicted by their rude attempts to afford relief. We should, however, approach the labours of Dr. Denman with feelings of gratitude, and state our disapproval of his opinions without any approach to asperity. Let us not intimidate others from throwing their mite into the general store of improvement, by casting undue severity upon one to whom we are so much indebted.

From what edition of Dr. Denman's Aphorisms our author quotes, we know not, but we believe the following extract to be inaccurate; and the criticism which arises from it, consequently uncalled for. In speaking of Dr. Denman's Aphorisms, Dr. Dewees remarks—

"His fifth (aphorism) declares 'it is meant, when the forceps are used, to supply with them the insufficiency or the want of pains.' Here is a plain and positive direction, one that the common sense of all mankind would at once agree is sound and proper, and one that would justify any body, in the absence of sufficient or efficient pains, to employ the forceps, and thus supply the deficiency of the natural powers; but all this prudent and well-tested direction is instantly destroyed by the next member of the aphorism,—namely, 'but, so long as the pains continue, we have reason to hope they will produce their effect, and shall be justified in waiting.'

"If this aphorism collectively has one particle of meaning in it, it forbids the use of the forceps so long as there are any pains, however feeble, transitory, and insufficient they may be for the end proposed: the value of pains must be estimated by their power upon the child to be moved, and not by the suffering the woman herself may endure. But let it be recollected that, beside the risk the child is running by its long delay in the passage, the soft parts of the mother are suffering from the long pressure of the child's head, subjecting them to contusion, inflammation, sloughing, &c.; and this to comply with a prejudice against the employment of the forceps, and that at a seasonable and proper time." (P. 303, 304.)

We have not the Aphorisms of Dr. Denman at hand, but, upon reference to the sixth edition of his "Introduction to Midwifery," we find it clearly stated, at page 254, that, "so long as the natural pains continue *with any degree of vigour*, there is always reason to hope that they will ultimately accomplish the effect of expelling the child without any artificial assistance, in which case the use of the forceps is not required." Let any man read this passage with an unprejudiced mind,—with a disposition to understand, not to misunderstand, the author, and he must acknowledge the rule of conduct is explicitly laid down for any practitioner, and that the opinion it inculcates is founded upon rational principles. It would be hyp[illegible]critical to object, that the term "with any degree of vigour" conveys no positive idea. It is very clear that the doctrine the above passage implies is, that, so long as we have reason to believe that the uterine action will effect delivery, instruments are not required.

Upon reference to page 253 of the work just quoted, it will appear that Dr. Denman was as sensible as our author of the necessity of not delaying the application of the forceps until the powers of the woman were so far exhausted as to threaten her safety. It is to combat an evil which has no longer existence,—to wage an unnecessary war of words, to enter into a formal statement of the impropriety of such injudicious procrastination. We have seen several cases which induce us to accede to the following opinion:

> "It is by no means unusual for the pains to cease after the application of the forceps, and that we are obliged to perform the delivery without their aid. We are at a loss to account for this; for it is contrary to what might reasonably be supposed. When, however, they continue with even a moderate force, we have for many years been in the habit of disengaging the instruments, and ceasing to draw when the head is about to pass through the external parts, that they may be the better supported, and the risk of laceration diminished. Should there be no pain, we are then constrained to continue our efforts until the head is without.
>
> "In removing the forceps before the head is delivered, we are aware we are departing from high authority; for Dr. Denman lays it down as a rule, that, 'in every case in which the forceps have been applied, they are not to be removed before the head is extracted, even though we might have little or no occasion for them." But, notwithstanding this positive injunction, we are entirely persuaded, from our own experience, it is the safer practice, if we regard the integrity of the soft parts of the mother worth preserving." (P. 311, 312.)

We confess we know nothing, from our own experience, of the use of the long forceps, when the head is above the superior aperture of the pelvis. The following objections to their employment suggest themselves, upon consideration of the subject. We conceive it must be always a very difficult task to grasp the head at all in that situation; that the direction in which the blades of the instrument are applied, when we do succeed, must be very uncertain, and that, consequently, the child will be in danger of considerable injury; that the probability of including some of the soft parts of the mother must be very great; and that, when this instrument has been applied in a *narrow* pelvis. the life of the child has been almost invariably lost. We differ entirely from Dr. Dewees in his opinion "that the long French forceps are the most preferable for even the unskilful." Ignorance and temerity are frequent associates; and we firmly believe that, if ever the long forceps come into general use, the most lamentable consequences would not unfrequently result.

With respect to the use of the instrument when the head is above the superior aperture, Dr. D. observes, that

"Smellie appears to be the first who had either sufficient skill or sufficient hardihood to apply the forceps while the head was free above the superior strait; and since his time he has had but few followers. This, however, has not risen so much from the contemplation of its dangers, as the consciousness of its difficulties. To employ them under such circumstances with success, it is necessary that the operator should be aware of all he might encounter in his enterprise, as well as to be well skilled in their application in the situations we have just been considering: therefore, it cannot be recommended as a resource to young or inexperienced practitioners." (P. 324.)

As a proof that the necessity for applying forceps with the head in the above named situation is very rare, the author states he has only been required to use them three times in thirty-five years. Even with Smellie himself, the original projector of this operation, the danger attending it appears to have been very great.

The rule of practice which has been transmitted from author to author for a long period, "that the forceps are never to be applied till the ear of the child has been within reach of the operator's finger for at least six hours, has undoubtedly many exceptions. Far be it from us to countenance the unnecessary and premature application of instruments; but it should be remembered that, if the forceps are properly used, and we are not called upon to restrict the use of any instrument, upon the mere assumption of its improper application, no harm can possibly arise to either the mother or child. We have known several instances in which the life of the patient has been endangered by submission to the above axiom. In our own practice, we have very frequently departed from it, and we have had no reason to doubt the propriety of our conduct.

Passing over several pages, which contain many well-known doctrines concerning breech and feet presentation, we arrive at a subject of much interest, "*of rigidity of the soft parts, as the cause of preternatural labour.*" This subject has been but imperfectly considered by the generality of writers on obstetrics. It is so common, that every practitioner must have been perplexed with it. It is true that time and suffering have generally overcome the difficulties created by this state; and therefore, perhaps, it has made but little impression. It is probably the most frequent cause of tedious labour. "We cannot pretend to point out the cause of this rigidity." Many interesting cases of rigidity, affecting different parts, are given. Bleeding largely is considered usually necessary. In this country, we apprehend that practitioners not unfrequently look upon such cases very supinely, and that every thing is left to nature, when she might fairly and effectually be assisted by art. It has been suggested by Dr. Elihu Smith, of New York, to employ the tobacco-glyster in such cases, from the similarity of effect produced by this remedy and copious bleeding. In one case, Dr. Dewees employed it: sickness, vomiting, and fainting, were produced, but the rigidity was not overcome. Bleeding *ad deliquium* was had recourse to, with the effect of producing relaxation, and the patient was delivered in a few minutes of a healthy child. The patient died on the sixth day.

"This case, notwithstanding its unfortunate termination, fully establishes the influence of blood-letting in this very distressing kind of rigidity, and proves its action to be different from that of tobacco, though the latter produces sickness, vomiting, and syncope. We do not think the slightest blame can attach to the bleeding; as the woman was very well until the sixth day, when diseases to which she was subject supervened, and carried her off." (P. 378.)

*On Uterine Hemorrhage.*—Dr. Dewees does not give a detailed account of the opinions of other writers upon this highly important subject, but pledges himself to a faithful selection of such opinions and observations as he considers most worthy of attention. The mode pursued is,

"First. To consider very briefly the nature of the connexion of the ovum with the internal face of the uterus.

"Secondly. To investigate the causes which may impair this connexion, and thus expose the source from which the blood is derived.

"Thirdly. To examine into the mode of action of these agents in effecting this lesion.

"Fourthly. To point out the several periods of utero-gestation at which this may take place; and trace the various consequences which may result from these periods.

"Fifthly. To notice the mode of treatment under the different stages and circumstances which may accompany the disease." (P. 382.)

We must pass briefly over the three first divisions, that we may take more space for the consideration of the effects produced by, and the treatment required for, uterine hemorrhage. The following observations merit attention, and, as they give the opinion of our experienced author upon two or three points which still divide us, we lay it before our readers.

"It may not be amiss to inquire how far we may have a control, or whether we have any, over uterine contraction, after it has once been called into action. The no small authority of Mr. Burns is against us when we say we think we have, though confessedly difficult of subjection. Yet, as it is a matter of high consequence to ascertain the truth upon this subject, we hope to be forgiven if we differ from that respectable writer. He says, 'when abortion is threatened, the process is very apt to go on to completion, and it is only by interposing *before the expulsive efforts are begun*, that we can be successful in preventing it; for, whenever the muscular contraction is *universally established, marked by regular pains, and attempts to extend the cervix and os uteri*, nothing, I believe, can check the process.'

"That it is a matter of uncertainty whether we succeed in our attempts to arrest uterine contraction after it is 'established,' must be acknowledged; but that it is never attended by success, we cannot concede: nor should the principle ever be inculcated, as it paralyses exertion, and withholds from the suffering female that comfort which the attempt rarely fails to give. Our own experience would, we think, in more instances than one, declare that we have been rewarded for the attempt to interrupt uterine contraction; and, should it fail nineteen times out of twenty, we are surely not justified in withholding the probable means. We, therefore, make it an invariable rule to treat the case as if we expected to meet with success, and have had no reason to suspect we are not doing our duty.

"There is one case, however, in which we never interfere, with the slightest prospect of a happy issue; and that is where the process of gestation has unequivocally ceased; and of which we take but one circumstance to be absolutely certain,—namely, where the breasts have become tender and tumid, and then pretty suddenly subside. It would here be a forlorn hope to administer remedies with a view of retaining the ovum.

"We are disposed to believe that this circumstance is the only one which marks the loss of life of the ovum with sufficient certainty: it is, perhaps, the only one that is unequivocal, since all others may be said to be deceptive. This mark was known to Hippocrates, and has, we believe, ever since his time, stood the test of experience. So long, then, as this sign is absent, we do not relax in our attempts to preserve the ovum. It must, however, be confessed, that we have known the ovum cast off where this symptom was wanting. Yet we are persuaded, in each of these instances, that the ovum preserved its vitality almost to the last moment, and that its expulsion was owing to the indomitable nature of the contractions of the uterus; and we think that this has obtained most generally with women who are in the habit of miscarrying. We do not stand alone in our opinion upon this subject." (P. 394, 395.)

It may be remarked, that the doctrine of Mr. Burns, and some other respectable authorities, which inculcates the necessity of "interposing before the expulsive efforts have begun," and the certainty of failure "when regular pains have occurred, and attempts made to distend the cervix and os uteri," is productive of a much greater evil than that "of paralysing the exertions" which would otherwise be used to prevent abortion. Under such circumstances, many practitioners are so convinced that abortion must ensue, that they do not confine themselves to remaining neutral, but they endeavour to hasten the event which they deem absolutely certain. For the reasons urged by Dr. Dewees, we hold this practice to be unjustifiable.

It is to be remembered, that neither pain nor hemorrhage, although each or both may be severe, *necessarily* produce abortion. In our own practice, we have seen some striking examples to the contrary. Where the "action of gestation" (to employ the language of Mr. Burns,) has ceased, it would be improper, or even prejudicial, to attempt the preservation of the ovum.

The treatment demanded for the prevention of abortion, when symptoms indicative of that accident occur, is clearly stated. One important point we must dwell upon for a moment. We have heard it stated, in a public medical discussion, by a respectable young lecturer on the practice of midwifery, that, "when the placenta is retained after the expulsion of the ovum, even in the first *three months* of pregnancy, it is not only practicable, but proper, to introduce the hand, or three or four fingers, into the uterus, and bring away the placenta." We look upon this as a very dangerous, and a very unjustifiable doctrine. We do not believe that the hand, or even two or three fingers, can be introduced into the uterus during the first three or four months of utero-gestation, in such a manner as to enable us to grasp the placental mass, and to withdraw it. It is true the uterus may be stimulated to contraction by the rude efforts to introduce the hand, and thus the placenta may be expelled. "At the early periods of pregnancy," says Dr. Dewees, "which are comprehended within the first five months, the uterine cavity is too small to admit the hand, or a couple of fingers, or even one; therefore, any attempt to deliver it by the hand alone will almost always fail. If this mass is entirely within the uterus, or even nearly so, the os uteri will be found most generally so much closed, even at the fifth month, as to prevent the introduction of the finger so as to hook down the placenta; and, as we descend from this to the second month or lower, it will be naturally so small as to prevent the intromission of even one." (P. 415.)

Sometimes a portion of the placenta may be felt without the os tincæ, and we may be enabled to extract the whole of it. Dr. D. has used, with the "most entire success," a small wire crotchet to bring away the placenta in the early months. The use of this instrument will require caution, and that our hands should be deliberately guided by our heads.

We reflect with much regret upon many cases, which have fallen within our notice, in which the unfortunate patient has been tortured for hours by the rough and cruel attempts of an ignorant practitioner to bring away the placenta during the early months of utero-gestation. We know, from the evidence of Dr. Denman,—and we may be permitted to add, from our own more limited experience,—that much less mischief will result from the retention of the placenta during the early periods of pregnancy, than from the medicines which are usually given to hasten its expulsion, or from manual assistance.

In the treatment of uterine hemorrhage during the first four months and a half, Dr. Dewees begins with the use of the acetate of lead, in large doses. "The acetate of lead should be given in doses and in frequency proportionate to the violence of the discharge. From two to three grains, guarded with opium, may be given every half hour, or less frequently, as circumstances may direct; or, in case the stomach be irritable, a very efficient mode of exhibiting it is per anum. Twenty or thirty grains may be dissolved in a gill of water, to which will be added one drachm of laudanum: this must be repeated *pro re nata.*" Dr. Merriman says, "the plumbi acetas, and many other internal remedies, which are efficacious in restraining chronic hemorrhagies, cannot be at all relied upon in the floodings of parturition. Such, we confess, has been our own opinion, but it has been founded more upon the weight of authority than our practical experience. We have much reliance upon the judgment and accuracy of Dr. Dewees, and are not at liberty to oppose *his* facts by *our* speculation. Dr. Merriman does not state whether his want of confidence in the remedy has been the result of his own experience.

With respect to the application of a plug into the vagina, during the first four months of pregnancy, which has excited so much contrariety of opinion, Dr. Dewees gives the following advice:—

"Whatever may be the rapidity of discharge in such cases, it is ever under command, so far as our experience will warrant the assertion, by the use of the tampon. It should be instantly resorted to, and its effects will be as quickly perceived. If the ovum can be preserved, we save a prodigious expenditure of blood: if it cannot, we not only do this, but obtain a most important truce, during which time nature achieves the separation and the final expulsion of it, without the further exhaustion of the patient; for Leroux tells us that, when the uterus is opened, the tampon is not only useful in stopping the discharge, but in stimulating the uterus to successful contraction." (P. 413.)

It is urgently recommended, and we conceive upon solid grounds, "that the ovum is never to be pierced before the commencement of the fifth month, unless the flooding is very profuse, the pains very urgent, and the os uteri pretty well opened." From the fifth month of pregnancy to the entire completion of the term of utero-gestation, the chances of a very profuse discharge are increased, from causes which are obvious, but which are, however, dwelt upon in detail, for the information of those who are mere students upon the subject. During this period the indications are precisely the same as during the "first period," but their fulfilment is not always effected after the same manner."

After having weighed the merits of the various modes of treatment which have been advised in uterine hemorrhage during the last months, the author thus describes the practice his experience tells him is the best:—

"In moderate uterine discharges, alum, the preparations of lead, digitalis, and the external application of cold, together with astringent injections per vaginam, &c. may very often succeed; and hence it is our uniform practice to exhibit the acetate of lead, either by the mouth or per anum (when the stomach is disturbed), in cases of this description: in a word, treating them in every respect as we should the mild ones in our 'first period.'

"But what reliance can be placed upon these comparatively feeble remedies in those cases of hemorrhage which threaten the life of the patient in a very short period of time; cases where the woman has been drained of by far the larger portion of her blood,—where there is syncope, convulsions, and an extinguished pulse? Can any man reconcile it to his conscience to stand by, waiting the success of a few grains of alum or of sugar of lead, or of a few drops of the tincture of foxglove, while the woman's life is rapidly passing away with the escaping blood? In such cases, success can only attend either of the two, or both, the other methods; and to these two we must direct the attention of the young practitioner in every case of a menacing appearance. Yet we are told of success attending the other, in some desperate instances.

"Of the effects of alum in severe cases, we can say nothing from our own experience; but, from what we have witnessed in those of a milder kind, we should not be tempted to place upon it much reliance: if given in small doses, it is insufficient to the end; and, when given in larger quantities, it has ever, in our hands, deranged the stomach so much as to be rejected; and of digitalis we can say nothing in any case.

"But, as this remedy is recommended by Mr. Burns for floodings accompanied with increased arterial action, it may deserve confidence to be placed in it; but for ourselves, we should not be much tempted to employ it,—not from its want of power over the circulating system, but from its general unmanageableness, and the permanency of depression it sometimes occasions.

"Of the sugar of lead we have a much higher opinion. This has been considered by some as a new remedy, but we find it was long since recommended by Etmuller, Friend, Kok, &c.; the two former in the form tinct. antiphthisic., and the latter in injections, combined with vinegar, per vaginam. Its effects are, for the most part, prompt and useful, and we constantly regard it as an important auxiliary. We have given it liberally, and often with the most decided advantage; and we very rarely fail to employ it in addition to our other means. It can be given by the mouth in the quantity already mentioned, or by injection, as before suggested.

"We have never, in cases of this kind, placed any reliance upon injections into the vagina, for several reasons:—1st, because they are very inconvenient in their exhibition, and especially as they must necessarily be rejected very quickly, and thus add to the discomfiture of the patient, by wetting, or rather floating her; 2d, because their effects are both uncertain and transient; 3d, because they may prove injurious by disturbing the patient, or by the removal of a useful coagulum. After delivery we have sometimes thought them useful, but never to the extent we are led to suppose by some.

"It is then our uniform practice, in every case of flooding during pregnancy of threatening aspect, or where, from the rapidity of the discharge, the woman's strength would quickly be exhausted, to use, in addition to the means just mentioned, the tampon. We have already said we have found fine sponge the best of any we have yet employed; but, where this cannot be procured, fine flax or very well picked tow, or old linen, may be substituted.

"When the latter substances are chosen, they should be used in portions of moderate size, and well moistened with sweet oil or melted lard; they should be introduced one by one, until the vagina is completely filled: the whole may be secured by a compress and T bandage. This latter precaution is not necessary when a sponge is used, if the piece be of proper size. It is introduced, from its compressibility, without the least inconvenience, being previously wetted with vinegar; and we believe it promotes coagulation quicker than any other substance we have hitherto employed, from its numerous cells quickly giving passage to the finer parts of the blood. It almost instantly puts a stop to the hemorrhage; and we are well persuaded, in some instances, we have been entirely indebted to it for the preservation of the woman's life.

"As this remedy is so confidently recommended by us, it may be well (as it will appear a novel one to many,) to say something more upon the subject, and endeavour to obviate the objections which have been urged against it by several respectable practitioners. The tampon is by no means a remedy of modern invention. It may be traced, as we are informed by Pasta, in several of the ancient authors; but Hoffman gave the first clear account of it, and it was used many years ago by Smellie. Leroux, however, is its great defender; and, coming from a man of his experience and candour, we felt at once a confidence in it, and first employed it upon the strength of his recommendation. He has given us many cases where its effects were very decidedly useful, and where it would seem, in all probability, that death would have been the inevitable consequence had it been omitted.

"It is truly a matter of surprise, nor are we able, upon any conjecture, to account for its not being considered by the British writers as a remedy in uterine hemorrhage, from the time of Smellie to that of Burns. It is true, indeed, it is mentioned by Dr. Denman, but he evidently places no reliance upon it: nor does Rigby lay the smallest stress upon its efficacy; he merely says, "that, should a case occur in which the uterus is too small to admit the hand, and yet the discharge so considerable as to endanger the life of the patient, before nature, by her own efforts, seems likely to effect an abortion, the method recommended by Leroux might, *I think,* with propriety be adopted.' Dr. Merriman merely mentions it *en passant*, and says he has 'had reason more than once to think it had been prejudicial;' but he mentions its employment only in hemorrhages succeeding the expulsion of the placenta. But Mr. Burns makes honourable mention of its efficacy, and seems to place no inconsiderable dependence upon it. Since the publication of his work upon Midwifery, others have regarded it as a valuable mean in arresting flooding; so that at this time it appears to have awakened more attention than it formerly did." (P. 418—422.)

The objections that have been urged against the tampon are maturely considered, and the observations of Dr. Dewees deserve attention. We must remember, however, in our treatment of this alarming disease, that "neither internal remedies nor external applications should be exclusively relied upon, longer than is decidedly consistent with the safety of the patient; for neither astringents of any kind, nor the tampon, can be availing in all cases, and, when they fail, there is but one resource,—namely, delivery." (P. 426.)

To affect artificial delivery, from the fifth to the sixth-and-a-half month, is not always possible. La Motte gives one example of this kind; Smellie another. Dr. Dewees has seen a similar failure. In our own practice, we have twice known the attempt made, and abandoned.

"It may be asked, what are we to do in cases of profuse hemorrhage, at any period from the fifth month to the full time, when the discharge threatens the life of the patient, and when the os uteri is both close and rigid? Are we to silently witness her death, rather than employ some violence to relieve her? Certainly not. If there really was no other remedy, forced delivery, with all its disastrous consequences, might be justifiable; but, as we have the power of plugging the vagina, and thus prevent the further issue of blood, we should have immediate recourse to it: and this plan, so far as we have witnessed, has not yet failed; and this experience is so supported by that of Leroux, as to entitle it to the utmost confidence. By this means, time is permitted to the natural agents of delivery for the performance of their duties, and this is done, for the most part, with both certainty and success." (P. 429.)

In opposition to the opinion of Dr. Denman, cordials are considered necessary in urgent cases.

Opium, as a remedy in uterine hemorrhage, has never, in the hands of Dr. Dewees, "merited the smallest commendation, or met with the slightest success." We hold this opinion to be too exclusively stated: there are certain highly irritable constitutions, in which the free use of opium will have the effect of restraining the hemorrhage.

The application of cold is undoubtedly carried too far in many instances. "When the pulse flags," says Dr. D. "and the woman is much exhausted, we not only forbid it, but pursue the opposite plan, by having a warm blanket, or other articles, to supply its place."

*Hemorrhage from the situation of the Placenta*, is next considered. We observe nothing in this section materially different to the well-known opinions of Dr. Rigby, which must be maturely studied by every man who is about to enter upon the practice of midwifery. Many pages are occupied in the minute consideration of the various kinds of uterine flooding, the attentive perusal of which we should recommend.

Chap. 36. *Of Puerperal Convulsions*.—Dr. Dewees gives but a brief sketch of his opinions upon this frightful malady. Some suppose puerperal convulsions arise from some peculiar irritation of the uterine fibre from pregnancy; others consider them truly epileptic; while others regard them as nervous or hysterical. "This difference in views necessarily leads to a difference in the mode of practice: the first makes safety to consist alone in immediate delivery; the second forbids the practice; while the third relies upon the use of opium. From what we have seen of this formidable complaint, we are persuaded that there is no one cause constantly operating to produce puerperal convulsions; nor is there any one mode of cure applicable to all." Dr. Dewees divides puerperal convulsions "into, 1st, epileptic; 2d, apoplectic; and 3d, hysterical; each of which may attack under two distinct conditions of the uterus, and requiring, from that circumstance, a difference of management." In the great majority of cases, we believe the distinctions proposed not to be applicable to practice. It will generally happen that we have the symptoms of hysteria and epilepsy so combined, that it would be impossible to determine to which form of disease the attack bears the greatest similitude. The distinction between hysteria and epilepsy, and between epilepsy and apoplexy, is not always an easy task at the bedside of the patient. The line of demarcation between these serious affections is more imaginary than real. If a patient suffers several paroxysms of puerperal convulsions, we shall probably have the hysterical form predominating in one,—then the epileptic,—and lastly the apoplectic; or, even during the same paroxysm, we shall have the characteristic signs of each of the above affections rapidly alternating with each other.

The symptoms which the author considers peculiar to each of the three species he enumerates, are minutely described. We do not find any mention, however, of a symptom which is frequently indicative of the approach of puerperal convulsions: we allude to a low singing, or rather humming of a few notes, as if the patient were endeavouring to recall some forgotten tune. We have, in several cases, found this symptom the first premonitory indication of approaching convulsions.

Several cases are given to show the treatment Dr. D. employs. It presents nothing peculiar: active bleeding and purgatives are the principal remedies. One case is detailed in which the patient was destroyed by the injudicious exhibition of opium. The following observation merits attention:—"In every case of convulsions, it is but too common for bystanders to oppose by strength the contractions of the agitated muscles. This practice cannot be too severely reprehended, as it is very injurious and most unnecessary: it subjects the patient to severe muscular pains, which last for very many days after the fits subside. All that should be done in such a case is to prevent the patient doing herself mischief, or prevent her throwing herself from the bed: a very moderate exertion is sufficient for this purpose, therefore violence should never be employed." (P. 511, 512.)

It is the opinion of some practitioners that, in those women who have been previously subject to hysteria, and who are of a highly irritable constitution, the treatment of puerperal convulsions may, and ought to be trusted to the free exhibition of opium. We have never seen so pure, so unmixed a case of the hysterical form, as to warrant us in adopting that practice. The authority of Dr. Merriman is important upon this subject. In his "Synopsis of difficult Parturition," he says, "of the use of opium I am not able to speak from experience, for I have never yet met with a case of puerperal convulsions, in which, at an early period of the disease, I could have dared to use the remedy." Dr. Hamilton has stated that he never saw a case where opium was given at the commencement, which did not terminate fatally.

We may observe, that, in a professed system of midwifery, we should have expected some observations upon the important and disputed question of the propriety of artificial delivery, in cases of puerperal convulsions. As it is not our business, however, to attempt an essay upon each subject which is merely touched upon by the author, we pass on with the mention of the deficiency.

The next chapter is important, but presents but little peculiar to the author: it treats *of the assisted Delivery of the Placenta*. In some cases the ergot is recommended.* "We may, however, remark, as a general rule, that the placenta is longer in descending where we cannot aid it by the cord, or where the

---

* An interesting detail of cases in which the ergot was given, is contained in the April Number (1823) of the *Annali Universali di Medicina*.—Rev.

cord is separated from it, than where it is strong and preserved: the reason of this is obvious. We should, therefore, in such cases promote the contraction of the uterus by frictions; and, from what we have experienced of the action of the ergot, we should be induced to give it a trial before we would pass the hand into the uterus: nor should we introduce the hand until we had satisfactorily proved it had failed in the object for which it was prescribed." (P. 525.)

In the 33d chapter, several cases of *Inversion of the Uterus* are detailed.

We have already drawn so largely upon our limits in our notice of Dr. Dewees, that we must pass over the remaining chapters with the mere enumeration of the subjects upon which they treat. Chap. 34, *of Twins, &c.* Chap. 35, *of the Rupture of the Uterus.* In opposition to the opinion of Dr. Denman and some other authorities, it is strongly contended that "it is almost always proper to interpose art in cases of ruptured uteri." There are many cases on record where the woman has been saved after a rupture of the uterus, in which the practitioner delivered by turning. The operation of gastrotomy is said sometimes to be necessary.

In the 36th chapter, the *management of labour where the pelvis is deformed* is considered. The operation of cephalotomy and the Cæsarean operation are considered. On the continent this last operation is resorted to in an early period of the labour, and the attempt to save both mother and child is sometimes crowned with the happiest result. It need scarcely be added, that, before so dreadful an operation is resorted to, the opinion of the most experienced practitioner should be consulted.

We have devoted much more space than usual to the consideration of the volume before us, not only from a deep feeling of the high importance of the various subjects upon which it treats, but from the respect with which we regard the labours of Dr. Dewees, who has passed so many years in the zealous execution of obstetrical duties. It is very true that this book contains much that has been said before: it would be unreasonable to expect every page to teem with novelty, when the subjects under discussion have for centuries attracted the mature deliberation of the skilful of every country. [redacted] Even the repetition of the doctrines of others by a veteran in practice, is highly valuable. By the slow accumulation of collective authorities, doubts are converted into certainties; and in every case we shall act with additional confidence, when we feel we are supported by the united opinion of many. 28 pages.

