# Errata in Our Number for August

**Published:** 1825-10

**Authors:** 


ERRATA *in our Number for August.*

Page 158, for "hypocritical" read "hype rcritical."

— 169, omit the words "which novelty could only be gained at the expence of accuracy."

